# Depletion of IgG-producing plasma cells in the colon by treatment of proteasome inhibitor ameliorates chronic DSS-induced colitis in mice

**DOI:** 10.1038/s41598-025-34868-6

**Published:** 2026-01-08

**Authors:** Shin Ebihara, Yukari Kimoto, Rumi Katsumoto, Noriko Konishi

**Affiliations:** 1 Biological/Pharmacological Research Laboratories, Takatsuki Research Center, Central Pharmaceutical Research Institute, Japan Tobacco, Inc, Osaka, Japan; 2 Department of Nutrition, Graduate School of Human Life and Ecology, Osaka Metropolitan University, Osaka, Japan; 3 Department of Clinical Immunology and Rheumatology, Tohoku University Graduate School of Medicine, Sendai, Miyagi Japan

**Keywords:** IgG-producing plasma cells, Chronic DSS-induced colitis model, Proteasome inhibitor, pANCA, Ulcerative colitis, Diseases, Gastroenterology, Immunology

## Abstract

Basal plasmacytosis is a histopathological hallmark of ulcerative colitis (UC). However, the precise roles of plasma cells in UC pathogenesis remain unknown. In this study, we investigated the effects of a proteasome inhibitor, which depletes plasma cells, on chronic colitis progression in mice to clarify their contribution to disease pathogenesis. Chronic colitis was induced in female C57BL/6 mice by three cycles of ad libitum dextran sulfate sodium (DSS) feeding followed by distilled water (DW), each cycle lasting seven days. The proteasome inhibitor bortezomib was administered intravenously twice weekly for three weeks after the second DSS/DW cycle. Elevated plasma IgG and perinuclear anti-neutrophil cytoplasmic antibody (pANCA) levels, as well as infiltration of IgG-producing plasma cells into the colon, were observed after the second DSS/DW cycle in chronic DSS-induced colitis model mice. Plasma cells in the colon exhibited an immature CD19⁺CD138⁺ phenotype. Bortezomib significantly ameliorated colitis and intestinal fibrosis by reducing plasma IgG and pANCA levels and the number of IgG-producing plasma cells in the colon. In conclusion, IgG-producing plasma cells were involved in colitis pathogenesis, and their depletion ameliorated colitis. Thus, IgG-producing plasma cells are associated with colitis pathogenesis and potential therapeutic targets for UC.

**Supplementary Information:**

The online version contains supplementary material available at 10.1038/s41598-025-34868-6.

## Introduction

Inflammatory bowel disease (IBD), including ulcerative colitis (UC) and Crohn’s disease, is a group of chronic relapsing gastrointestinal tract disorders pathologically characterized by intestinal inflammation and epithelial injury^1,2^. Recently, a “treat-to-target” approach has been proposed for mucosal healing^3^. However, the efficacy of many drugs used in clinical therapies remains inadequate^4^. Basal plasmacytosis, defined as dense infiltration of plasma cells around the deep part of the lamina propria (LP) or at the base of colon crypts, is a histological feature of IBD, particularly UC^5,6^. High degree of basal plasmacytosis is associated with high clinical relapse rate^7,8^, shorter clinical relapse time^9^, and elevated fecal calprotectin levels (disease activity markers) in patients with UC^10^.

For patients with UC, several antibodies are used as serological markers^11^. Specifically, perinuclear anti-neutrophil cytoplasmic antibody (pANCA), an autoantibody, is the best marker for UC in terms of sensitivity and specificity^11^. Recently, autoantigens, such as integrin αvβ6^12^ and endothelial protein C receptor^13^, have also been identified in patients with UC. Serum levels of IgG^14^ and pANCA^15^ positively correlate with the Mayo Clinic score, suggesting that IgG-producing plasma cells are associated with UC pathogenesis. Plasma cells comprised one of the major cellular components in the colonic LP of patients with UC^16−18^. In a healthy state, most plasma cells are IgA-producing plasma cells; however, marked IgG-producing plasma cell infiltration is observed in the colon of patients with UC^17,19^. IgG-producing plasma cells infiltrate the inflamed mucosa and critically influence UC pathogenesis by exacerbating mucosal inflammation by activation of pathogenic intestinal macrophages via Fc gamma receptor (FcγR) signaling^17^. Despite these insights, the precise roles of IgG-producing plasma cells in UC remain unclear^17,19^.

Chronic dextran sulfate sodium (DSS)-induced colitis model is established by administering three repeated cycles of *ad libitum* of DSS followed by distilled water (DW) every seven days^20,21^. The chronic DSS-induced colitis model exhibits more resemblance to chronic UC than the acute DSS-induced colitis model in terms of the association with both myeloid cells, such as macrophages and neutrophils, and lymphocytes, such as T and B cells^22^. Therefore, the chronic colitis model is often used to evaluate the therapeutic efficacy of anti-inflammatory drugs for UC, such as glucocorticoids (dexamethasone)^23^, Janus kinase (JAK) inhibitors (tofacitinib)^24^, and anti-tumor necrosis factor (TNF)-α antibodies^23,25^.

Bortezomib is a proteasome inhibitor that enhances the accumulation of unfolded proteins with the induction of endoplasmic reticulum (ER) stress, leading to apoptotic cell death^26^. Plasma cells synthesizing high levels of immunoglobulins exhibit relatively high sensitivity to bortezomib^27^, contributing to the clinical success of bortezomib for multiple myeloma treatment^28^. Bortezomib ameliorates lupus nephritis^29^, ANCA-induced glomerulonephritis^30^, and myasthenia gravis (MG)^31^ in rodents by inhibiting the production of autoantibodies by plasma cell depletion. Additionally, bortezomib protects against autoimmune diseases, such as systemic lupus erythematosus^32^, thrombotic thrombocytopenic purpura^33^, MG^34^, and neuromyelitis optica spectrum disorder^35^, by reducing the plasma cell number and suppressing autoantibody production.

In this study, we established a chronic colitis model by repeated DSS treatment of mice and investigated the roles of IgG-producing plasma cells in colitis pathogenesis. Additionally, to demonstrate plasma cells as causative cells for chronic colitis, we examined the effects of bortezomib, which depletes plasma cells, in a colitis model.

## Results

### pANCA and anti-flagellin IgG are produced during intestinal inflammation in chronic DSS-induced colitis model mice

**Fig. 1:**
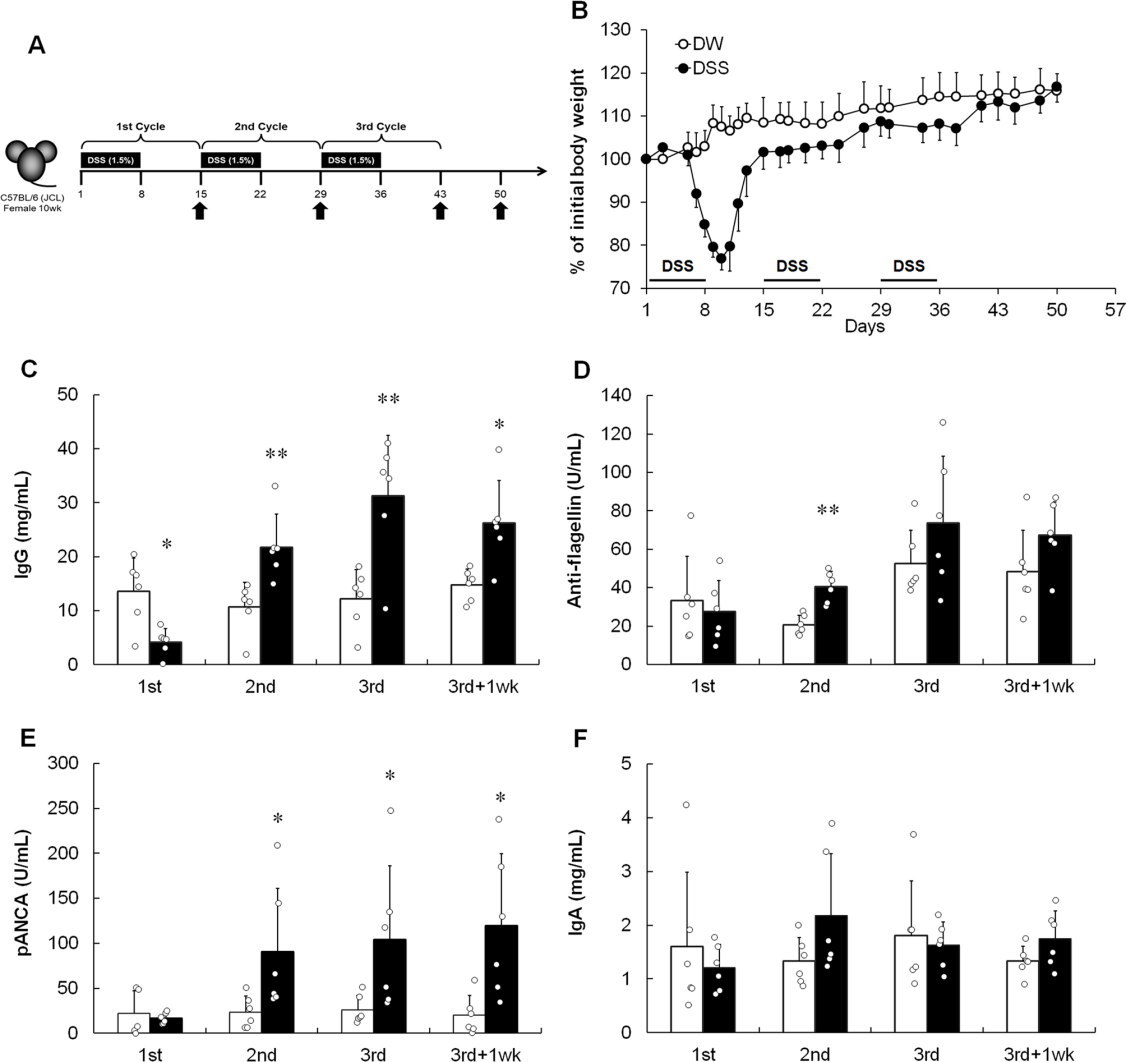
Involvement of immunoglobulin production in chronic DSS-induced colitis. (**A**) Experimental protocol. Mice were fed with three cycles of 1.5% DSS for seven days followed by DW *ad libitum* for seven days, after which they were administered DW for seven days. Arrows indicate the analysis time. (**B**) Relative weight curve of the DW (open circle)- and DSS (closed circle)-treated groups. Data are represented as mean ± SD. *n* = 10 animals per group. (**C**) Total IgG, (**D**) anti-flagellin IgG, (**E**) pANCA, and (**F**) total IgA levels measured by ELISA. Results are shown as mean ± SD with individual data. *n* = 6 animals per group. *p < 0.05 and **p < 0.01 vs. DW-treated group determined by Student’s or Aspin–Welch *t*-test.

Chronic colitis was induced by administering three repeated cycles of DSS. Each cycle involved the administration of 1.5% DSS for seven days, followed by sterile DW *ad libitum* for seven days (Fig. 1A). In the DSS-induced colitis model, mice treated with DSS started to exhibit clinical signs of colitis, including weight loss with loose stools, diarrhea, and hematochezia, after five days of DSS administration during the first DSS/DW cycle. Body weight further decreased when DW was reintroduced on day 8, with maximal weight loss observed on day 10 (Fig. 1B). Colitis model mice started to regain weight at the end of the first DSS/DW cycle (day 15) until the initial body weight was reached at the end of the experiment. As shown in Fig. 1C, after the first DSS/DW cycle, during the course of acute intestinal inflammation, total IgG levels in the plasma were lower than those in DW-treated mice, consistent with a previous report^36^. However, after the second DSS/DW cycle during the chronic inflammation phase, total IgG levels were higher than those in DW-treated mice (Fig. 1C). Next, we examined whether IgG against flagellin, protein subunit of bacterial flagellum, or myeloperoxidase (MPO), an autoantigen, were present in the chronic DSS-induced colitis model mice during intestinal inflammation. As shown in Fig. 1D, although the plasma flagellin-specific IgG antibody levels increased only after the second DSS/DW cycle (day 29), consistent with previous reports^36,37^, these antibody levels did not increase during the chronic phase. In contrast, pANCA (anti-MPO IgG) levels consecutively increased after the second DSS/DW cycle until the experimental endpoint (day 50; Fig. 1E). Plasma IgA levels did not increase at any time point, indicating that they did not affect colitis development (Fig. 1F). Taken together, these results suggest that autoantibodies, such as pANCA, are involved in colitis pathogenesis.

## IgG-producing plasma cells infiltrate into colonic LP in chronic DSS-induced colitis model mice

**Fig. 2:**
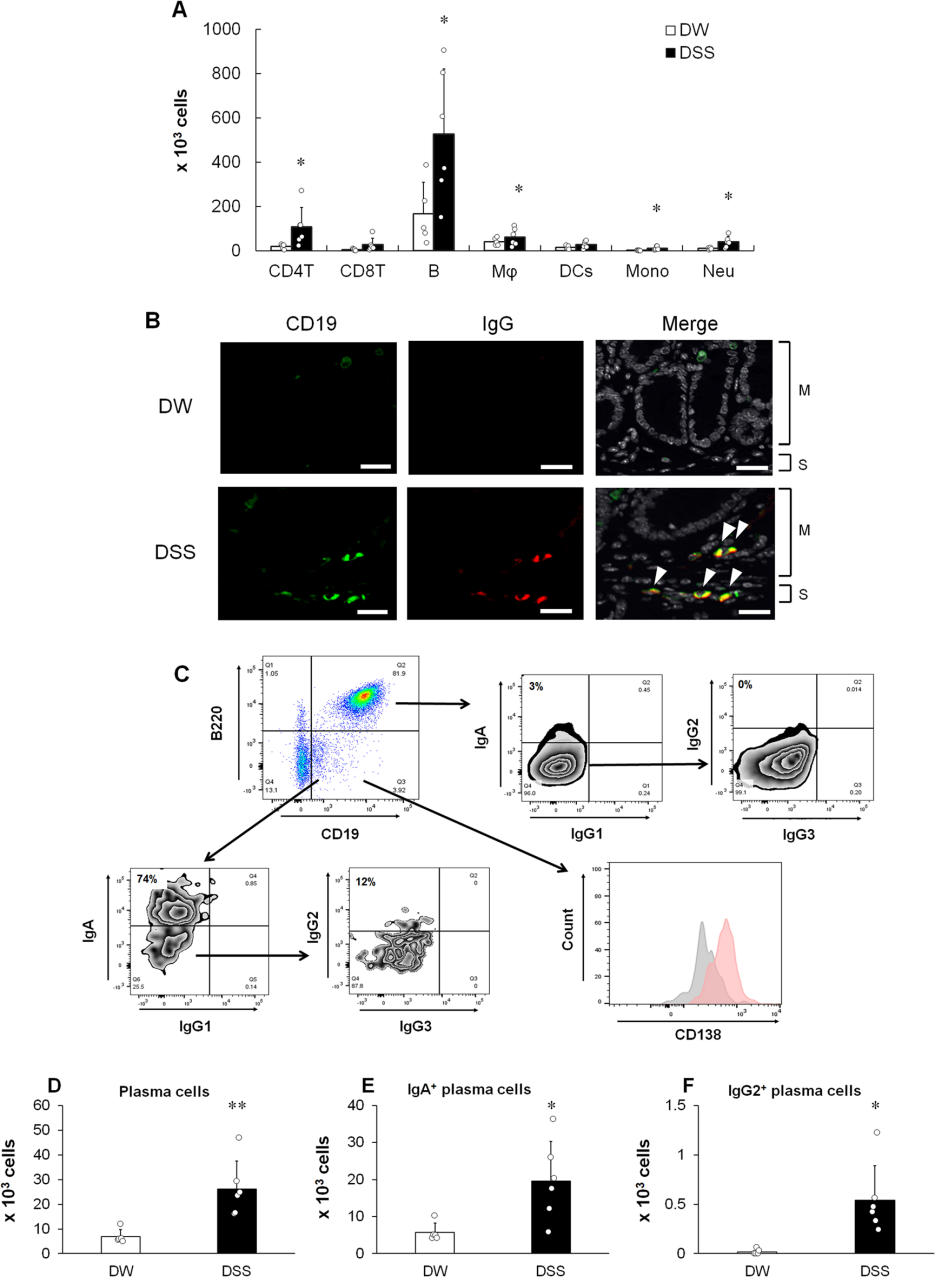
Association of IgG-producing plasma cells with colitis onset. (**A**) Numbers of infiltrating inflammatory cells, including CD4 T cells (CD4T), CD8 T cells (CD8T), B cells (B), macrophages (Mφ), dendritic cells (DCs), inflammatory monocytes (Mono), and neutrophils (Neu), in DW (white column)- and DSS (black column)-treated groups at the experimental endpoint (day 50) determined by flow cytometry. (**B**) Immunofluorescence double staining of the colon of DW- and DSS-treated groups with anti-CD19 (green) and anti-IgG (red) at the experimental endpoint. DAPI (white). Double-positive cells displaying CD19^+^IgG^+^ are observed in yellow (arrowheads). Data are representative of five mice per group. M: Mucosa; S: Submucosa. Scale bar, 25 μm. (**C**) Intracellular immunoglobulin staining of B220^−^CD19^+^ immature plasma cells and B220^+^CD19^+^ B cells at the experimental endpoint. In the expression analysis of CD138, gray histogram indicates the isotype control staining. Data are representative of five DSS-treated mice. (**D**) Total plasma, (**E**) IgA-producing, and (**F**) IgG2-producing plasma cell numbers were determined. (**A, D, E**, and **F**) Results are shown as mean ± SD with individual data. *n* = 5–6 animals per group. *p < 0.05 and **p < 0.01 vs. DW-treated group determined by Student’s or Aspin–Welch *t*-test.

Flow cytometric analysis revealed significantly higher numbers of CD4 T cells (TCRβ^+^CD335^−^CD4^+^), B cells (CD19^+^B220^+^), macrophages (CD11b^+^F4/80^+^), inflammatory monocytes (CD11b^+^F4/80^−^Ly6C^+^), and neutrophils (CD11b^+^Ly6G^+^) in the colonic LP of DSS-treated mice than in that of DW-treated mice at the experimental endpoint (Fig. 2A and Supplementary Fig. S1). Furthermore, consistent with the increase in total plasma IgG and pANCA production (Fig. 1C and E), IgG-expressing CD19^+^ cells were observed in the mucosa and submucosa of the colon of DSS-treated mice at the experimental endpoint (Fig. 2B). We confirmed the infiltration of CD19^+^ cells into the colonic LP of the chronic DSS-induced colitis model using flow cytometry. CD19⁺ cells infiltrating the colon consisted of B220⁺CD19⁺ B cells and B220⁻CD19⁺CD138⁺ plasma cells. Although plasmablasts have previously been reported to exhibit a B220⁺ phenotype in mice^38^, the B220⁻CD19⁺CD138⁺ population identified in this study appears to represent a distinct subset in the colon of the chronic DSS-induced colitis model. Given that CD19⁺CD138⁺ plasma cells are defined as immature plasma cells in the colon of patients with UC^17,39,40^, this B220⁻CD19⁺CD138⁺ subset likely represents the murine counterpart of immature plasma cells (Fig. 2C). Low CD138 levels in immature plasma cells are possibly due to its instability, which affects its detection during collagenase digestion^41^. Here, immature plasma cells expressed IgA and IgG2 (Fig. 2C). In contrast, B cells mildly expressed IgA, but not IgG (Fig. 2C). In addition to the various inflammatory cells, the number of immature plasma cells also increased in the colon of the chronic DSS-induced colitis model (Fig. 2D). As shown in Fig. 2E and F, both IgA- and IgG-producing immature plasma cells were more abundant in DSS-treated mice than in the DW-treated mice. Overall, plasma cells infiltrating the colon in the chronic DSS-induced colitis model were IgG2-producing and exhibited an immature phenotype.

## Effects of bortezomib on the inflammatory responses in chronic DSS-induced colitis model mice

**Fig. 3:**
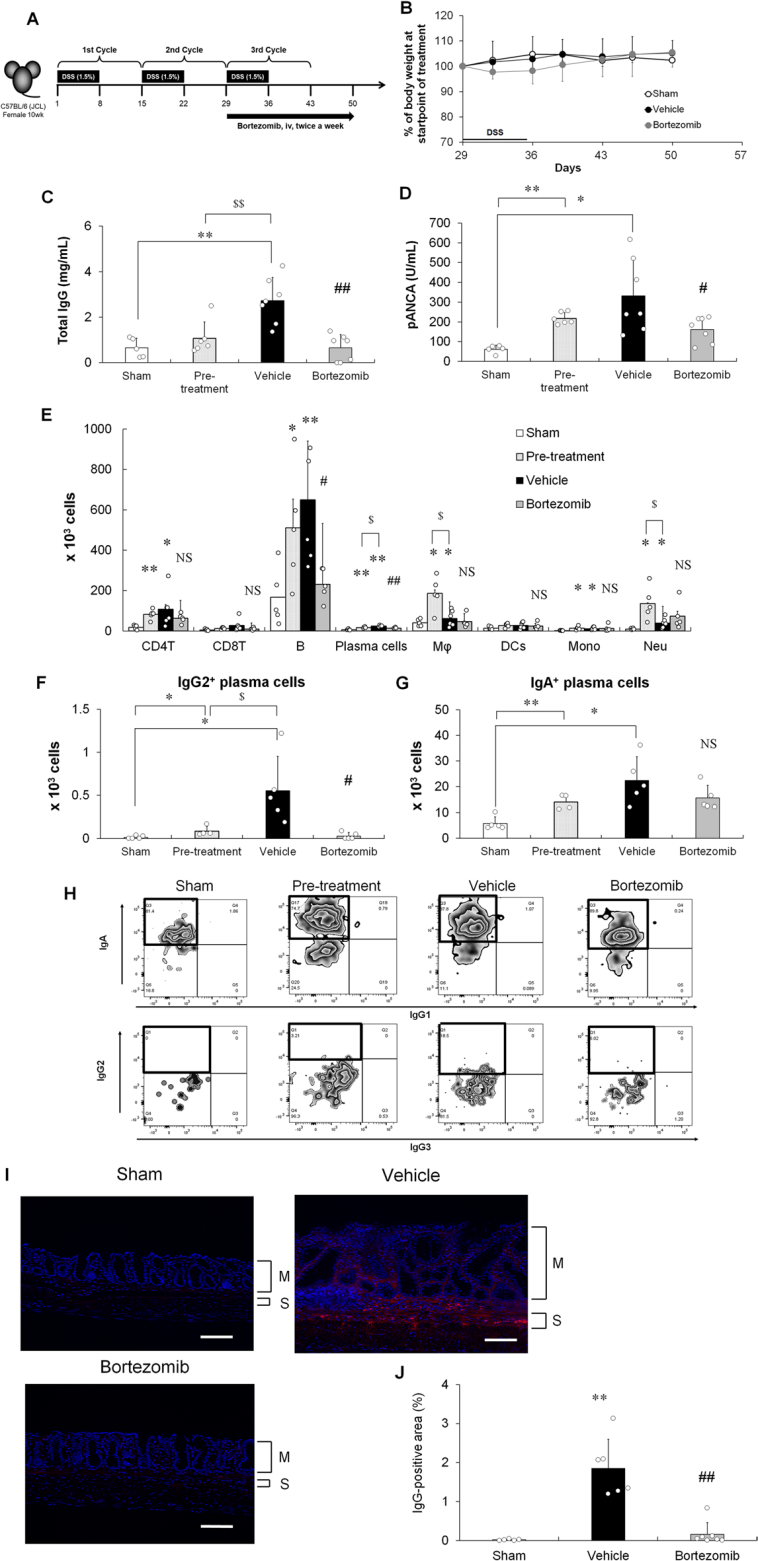
Effects of bortezomib on plasma IgG levels and IgG-producing immature plasma cells in the colon of chronic DSS-induced colitis model mice. (**A**) Experimental design of bortezomib administration process. Arrow indicates the duration of bortezomib administration. (**B**) Relative weight curve of sham (open circle)-, vehicle (black circle)-, or bortezomib (grey circle)-treated groups. (**C**) Total IgG and (**D**) pANCA levels measured by ELISA. (**E**) Numbers of infiltrating inflammatory cells, including CD4 T cells (CD4T), CD8 T cells (CD8T), B cells (B), plasma cells, macrophages (Mφ), dendritic cells (DCs), inflammatory monocytes (Mono), and neutrophils (Neu), in sham-treated (white column), pre-treatment (dotted column), vehicle-treated (black column), and bortezomib-treated (grey column) groups determined by flow cytometry. (**F**) IgG2- and (**G**) IgA-producing immature plasma cell number were determined by flow cytometry. (**H**) Representative flow cytometric plots of sham-treated (white column), pre-treatment (dotted column), vehicle-treated (black column), and bortezomib-treated (grey column) groups. The areas enclosed by the black squares indicate immature plasma cells producing IgA (upper panel) or IgG2 (lower panel). (**I**) Representative microphotographs of IgG deposits in the colonic tissues of sham-, vehicle-, and bortezomib-treated mice. IgG (red); DAPI (blue). Scale bar, 100 μm. (J) Percentage of IgG-positive area in the distal colon. M: Mucosa; S: Submucosa. (**B** − **F** and **H**) Results are shown as mean ± SD with individual data. *p < 0.05 and **p < 0.01 vs. sham-treated group; §*p* < 0.05 and §§*p* < 0.01 vs. pre-treatment group; #*p* < 0.05 and ##*p* < 0.01 vs. vehicle-treated group (Student’s or Aspin–Welch *t*-test). NS: not significant vs. vehicle-treated group (Student’s or Aspin–Welch *t*-test). *n* = 5–7 animals per group.

To investigate the role of colon-infiltrating IgG-producing immature plasma cells in the chronic colitis progression, immature plasma cells were depleted by systemic administration of bortezomib. For intervention studies, bortezomib was administered twice weekly to the mice at the end of the second DSS/DW cycle (Fig. 3A). No significant weight loss was observed during the period of bortezomib administration, suggesting that the treatment did not cause apparent systemic toxicity (Fig. 3B). To explore the inhibitory effects of bortezomib, total IgG and pANCA levels in plasma, which were upregulated during colitis progression, were examined. Bortezomib significantly decreased the total IgG (Fig. 3C) and pANCA (Fig. 3D) levels at the experimental endpoint comparable to those at the start of treatment (pre-treatment). As shown in Fig. 3E, the numbers of CD4 T cells, B cells, plasma cells, macrophages, inflammatory monocytes, and neutrophils increased at the start of bortezomib treatment. Moreover, the numbers of macrophages and neutrophils decreased, but those of other cells, excluding plasma cells, remained unchanged at the end of treatment in the vehicle group. Bortezomib significantly decreased the numbers of B and plasma cells, but not CD4 T cells, macrophages, inflammatory monocytes, and neutrophils, in the colonic LP (Fig. 3E). As shown in Supplementary Figure S2, the production of inflammatory cytokines, such as TNF-α, interleukin (IL)-6, and IL-1β, and nuclear factor (NF)-κB activation increased at the pre-treatment stage. Moreover, these cytokine productions and NF-κB activation decreased by the end of treatment in the vehicle group. Bortezomib had no effects on these cytokine productions and NF-κB activation (Supplementary Fig. S2). As shown in Fig. 3F − H, the numbers of IgG2- and IgA-producing immature plasma cells increased during colitis progression. Notably, bortezomib induced a more pronounced reduction in IgG2-producing immature plasma cells than in total B cells or total plasma cells, but not IgA-producing immature plasma cells, in the colonic LP (Fig. 3F and G). Moreover, IgG was deposited in the mucosa and submucosa of the colon in the chronic DSS-induced colitis model at the experimental endpoint, similar to that observed in patients with UC (Fig. 3I)^42^. Bortezomib significantly decreased the colonic deposition of IgG (Fig. 3I and J).

## Amelioration of colitis and intestinal fibrosis by bortezomib in chronic DSS-induced colitis model mice

**Fig. 4:**
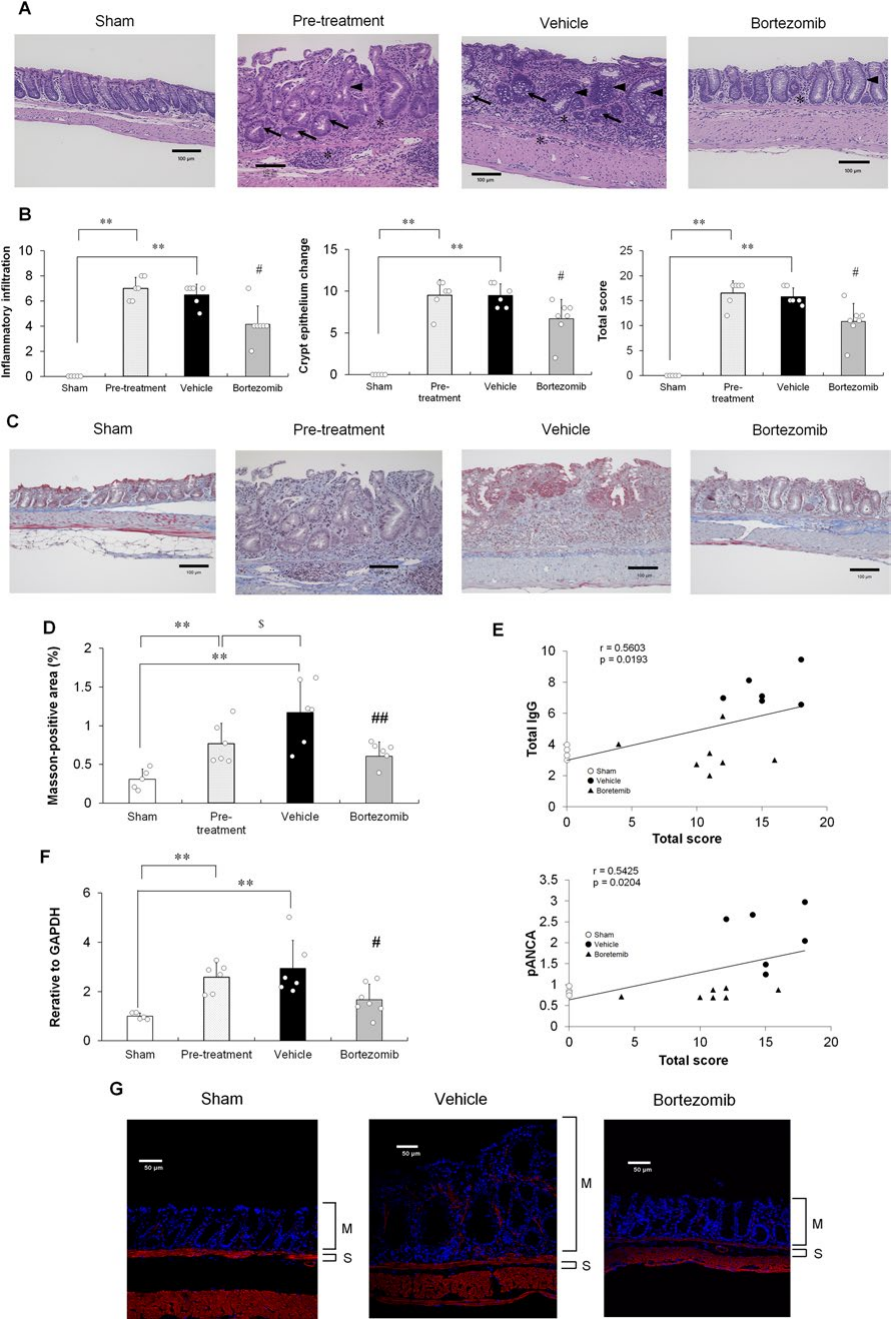
Effects of bortezomib on colitis progression and intestinal fibrosis in the distal colon of chronic DSS-induced colitis model mice. (**A**) Representative microphotographs of the colonic tissues of sham-treated, pre-treatment, vehicle-treated, and bortezomib-treated mice (H&E staining). Pre-treatment: baseline for treatment of vehicle and bortezomib. Immune cell infiltration (asterisk), epithelial hyperplasia (arrowhead) and goblet cell and crypt loss (arrow). Scale bar, 100 μm. (**B**) Histological scores of inflammatory infiltration, crypt epithelial changes including epithelial hyperplasia, goblet cell loss, crypt loss, cryptitis, and crypt abscess and total score at the experimental end point. Results are shown as mean ± SD with individual data. **p < 0.01 vs. sham-treated group; #*p* < 0.05 vs. vehicle-treated group (Mann–Whitney *U* test). *n* = 5–7 animals per group. (**C**) Representative microphotographs of the colonic tissues of sham-treated, pre-treatment, vehicle-treated, and bortezomib-treated mice (Masson’s trichrome staining). Scale bar, 100 μm. (**D**) Percentage of Masson-positive area (blue area) in the distal colon. **p < 0.01 vs. sham-treated group (Aspin–Welch *t*-test). Results are shown as mean ± SD with individual data. §*p* < 0.05 vs. pre-treatment group (Student’s *t*-test). ##*p* < 0.01 vs. vehicle-treated group (Student’s *t*-test). *n* = 5–7 animals per group. (**E**) Correlation of histological score and plasma IgG levels or pANCA levels. Sham- [open circle], vehicle- [closed circle], and bortezomib-treated [closed triangle] groups. *n* = 5–7 animals per group. (**F**) Transcript level of *Tgfβ1*. Results are shown as mean ± SD with individual data. **p < 0.01 via Aspin–Welch *t*-test vs. sham-treated group. #, *p* < 0.05 by Student’s *t*-test performed versus vehicle-treated group. (**G**) Representative microphotographs of the colonic tissues of sham-, vehicle-, and bortezomib-treated mice at the experimental endpoint. αSMA (red); DAPI (blue). M: Mucosa; S: Submucosa. Scale bar, 50 μm.

To investigate the effects of bortezomib on colitis pathogenesis and intestinal fibrosis, we performed histopathological evaluation of colitis (H&E staining)^43^ and quantification of collagen deposition in the mucosa and submucosa of the colon (Masson’s trichrome staining)^44,45^. At the start of treatment with bortezomib (pre-treatment), the mice already exhibited colitis (Fig. 4A and B) and intestinal fibrosis (Fig. 4C and D). Bortezomib ameliorated colitis (Fig. 4A and B) and inhibited intestinal fibrosis progression (Fig. 4C and D), maintaining the condition at approximately the baseline level. As shown in Supplementary Figure S3A–C, bortezomib significantly reduced the colon weight-to-length ratio and colonoscopic disease severity, including mucosal thickening, vasculature, and granularity, at the experimental endpoint. As shown in Fig. 4E, the significant correlations existed between total IgG and pANCA levels in plasma and total histological score, suggesting that total IgG and pANCA levels in plasma may be essential for the pathogenesis of colitis in the DSS-induced colitis model. The expression of fibrosis-related markers, TGFβ at mRNA level was also increased from the start of treatment to the experimental endpoint [Figure 4F]. Moreover, the treatment of bortezomib reduced the expression of TGFβ at mRNA levels [Figure 4F]. As shown in Fig. 4G, alpha-smooth muscle actin (αSMA) was localized not only in the typical tissue layers, such as muscularis mucosa and muscularis propria, but also in the mucosa of the chronic DSS-induced colitis model, similar to that observed in patients with UC^46^. Bortezomib decreased the αSMA levels in the colon at the experimental endpoint (Fig. 4G). Along with the cell count results shown in Fig. 3E–G, these findings suggest that bortezomib ameliorates colitis and the intestinal fibrosis progression by selectively depleting the IgG-producing plasma cells, without significantly affecting the other immune cells, including innate immune and CD4 T cells. Furthermore, anti-inflammatory agents targeting the innate immune or T cells, such as glucocorticoids (prednisolone), JAK inhibitors (tofacitinib), and anti-TNF-α antibodies, did not inhibit the development of colitis in the chronic DSS-induced colitis model (Supplementary Fig. S4). Therefore, IgG-producing immature plasma cells play crucial roles in the development and progression of colitis in the chronic DSS-induced colitis model. In summary, bortezomib significantly ameliorated chronic colitis by reducing the number of colonic IgG-producing immature plasma cells in the colon of the chronic DSS-induced colitis model.

## Discussion

In a double-blind, randomized, placebo-controlled trial (phase II trial), anti-CD20 antibody rituximab did not induce remission in moderately active UC^47^. Rituximab depleted B cells, but not plasmablasts/plasma cells, in the colonic mucosa of patients with UC in a clinical trial, considering that plasmablasts/plasma cells remaining at the site of inflammation continuously supported the ongoing inflammatory process of UC^48^.

In this study, we found that the chronic DSS-induced colitis model exhibits resemblance to the chronic nature of human UC in terms of histopathological features, including IgG-producing plasma cell infiltration into the colon. Interestingly, flow cytometric analysis revealed that the IgG-producing plasma cells infiltrating the colonic LP of chronic DSS-induced colitis model mice corresponded to the immature plasma cell subset reported in patients with UC, as previously described^17,39,40^. Antimicrobial antibodies are produced in the early stages, whereas autoantibodies are produced in the later stages of colitis. Therefore, colonic bacteria possibly contain proteins cross-reactive to the autoantigen epitope, resulting in a destructive inflammatory response triggered by the influx of luminal bacterial products into the gut wall via epithelial damage directed toward a self-antigen, leading to the progression from acute to chronic colitis in a colitis model, similar to that observed in patients with UC^49,50^.

This study showed that bortezomib, a plasma cell-targeted proteasome inhibitor, significantly improved the colonoscopic and histopathological manifestations in the chronic DSS-induced colitis model. In addition to inducing ER stress, bortezomib inhibits NF-κB activation^51^. Bortezomib also inhibits the production of inflammatory cytokine, such as IL-6, TNF-α, and IL-1β, by suppressing NF-κB activation, thereby ameliorating acute DSS-inducing colitis^52^. In this study, the cellular dynamics and cytokine profiles at the pre-treatment stage indicate that heightened activation of myeloid cell populations likely contributes to the progression of colitis during the acute phase, consistent with previous observations^52^. However, the cellular dynamics and cytokine profiles at the treatment stage suggested that the contribution of myeloid cells is limited during the period in which bortezomib was administered. In the therapeutic experiment, bortezomib did not suppress myeloid cell activation but instead reduced the numbers of B cells and total plasma cells, with a particularly pronounced effect on IgG-producing immature plasma cells. Bortezomib also reduced not only plasma IgG and ANCA levels but also IgG deposition in the colon of chronic DSS-induced colitis model mice. Therefore, bortezomib ameliorated colitis progression predominantly by reducing the number of IgG-producing immature plasma cells in the colon. Interestingly, bortezomib did not decrease the number of IgA-producing immature plasma cells in the colon, suggesting differential activation of the ER stress-induced unfolded protein response between IgA- and IgG-producing immature plasma cells. These differences may reflect distinct regulatory mechanisms, which need to be further investigated. As IgA production at mucosal sites is necessary to maintain a non-inflammatory state and prevent inflammatory responses^53^, targeting IgG-producing immature plasma cells rather than IgA-producing immature plasma cells is important to prevent colitis.

UC is associated with progressive fibrosis of the mucosa and submucosa linked to the severity and chronicity of inflammation^54,55^, suggesting the importance of deep remission, including histological remission, as a therapeutic target for UC^56^. Intestinal fibrosis was also observed in our chronic DSS-induced colitis model, consistent with previous reports^42,43^, was suppressed by bortezomib. In the model, TGF-β expression increased concomitantly with the development of fibrosis, and this elevation was effectively suppressed by bortezomib treatment. These results suggest that plasma cells may play a pivotal role in promoting fibrosis via TGF-β production, as described previously^57^. Bortezomib significantly reduces lung fibrosis in bleomycin-treated mice by depleting the plasma cells, indicating the causal role of plasma cells in lung fibrosis development^58^. Thus, these findings suggest plasma cells as therapeutic targets for fibrosis, including lung and intestinal fibrosis.

In contrast to previous reports [23 − 25], our chronic DSS-induced colitis model was resistant to treatment with glucocorticoids (prednisolone), JAK inhibitors (tofacitinib), and anti-TNF-α antibodies, possibly might be due to the variability in the microbiome among different facilities^59^. Nevertheless, the underlying mechanism responsible for this resistance remains to be elucidated. However, our chronic DSS-induced colitis model was possibly a severe UC murine model that was not responsive to major therapeutic agents, as described above. Serum pANCA levels are associated with the lack of response to anti-TNF-α therapy in patients with UC^60,61^. Moreover, number of infiltrating plasma cells in the colon is higher in patients with UC non-responsive to anti-TNF agents^62^ and glucocorticoids (prednisolone)^63^ than in the responders. Therefore, plasma cell depletion might serve as an effective therapeutic approach for drug-resistant UC characterized by marked plasma cell infiltration into the colon.

In conclusion, this study demonstrated the ameliorative effects of bortezomib, which depletes plasma cells, on chronic DSS-induced colitis. A limitation of this study is that it did not include direct functional experiments confirming whether the ameliorating effect of bortezomib is specifically mediated by the depletion of IgG-producing plasma cells. Future studies should address this limitation and further explore the therapeutic potential of safer and more selective plasma cell-targeting agents for UC. Nevertheless, this study provides insights into the in vivo functions of IgG-producing immature plasma cells in the colonic LP of patients with UC. Although derived from a preclinical animal model, our findings suggest that IgG-producing immature plasma cell depletion in the colon ameliorates chronic colitis, highlighting a novel therapeutic approach for human UC.

## Materials and methods

### Mice

Female C57BL/6J (B6J) mice (weighting 19 ± 2 g) were purchased from CLEA Japan (Tokyo, Japan) and maintained under specific pathogen-free conditions at a room temperature of 23 ± 3 °C and air humidity of 55 ± 15% under a 12/12 h light/dark cycle.

## Chronic DSS-induced colitis model establishment and treatment

Colitis was induced as previously described^20,21^. Briefly, mice were fed 1.5% DSS (36–50 kDa; MP Biomedicals, Solon, OH, USA) dissolved in sterile DW (Otsuka Pharmaceutical, Tokyo, Japan) *ad libitum* for seven days, followed by DW for seven days. This DSS/DW cycle was repeated twice, after which the mice were administered DW for seven days to establish a chronic DSS-induced colitis model. Sham group was treated with DW. Bortezomib was purchased from Santa Cruz (Dallas, TX, USA), dissolved in dimethyl sulfoxide (Nacalai Tesque, Kyoto, Japan) at a concentration of 10 mg/mL, and diluted 100-fold in PBS. In the therapeutic experiment, female B6J mice were mixed and caged into groups of seven mice at ten days from the initial day based on their body weight (bw). Then, the mice were intravenously treated with the vehicle alone (1% DMSO/PBS) or bortezomib (750 µg/kg bw^29,30^ twice weekly for three weeks from the end of the second DSS/DW cycle. In another experiment, after the second DSS/DW cycle, the mice were gavaged daily with another vehicle (0.5% methylcellulose (MC)), 3 mg/kg bw prednisolone or 100 mg/kg bw tofacitinib, or administrated intraperitoneally 1000 µg of anti-TNF-α antibody (clone TN3-19.12; Bio X cell, Lebanon, NH, USA) or Armenian hamster IgG (Bio X cell) twice a week.

### Colonoscopy and histology

For colonoscopy, the mice were anesthetized with isoflurane (Viatris, Tokyo, Japan), and feces were removed by injecting saline through a flexible feeding tube (Fuchigami, Kyoto, Japan). A rigid telescope (diameter, 1.9 mm; Smith and Nephew, London, UK) was rectally inserted into the mice up to 3 cm using the Olympus CLH-250 Xenon Light Source (Olympus, Tokyo, Japan), as previously described^60^. During endoscope withdrawal, a video of the distal colon was recorded using the Olympus Video System OTV-SC2 (Olympus) and TEAC UR-4MD Medical Video Recorder (TEAC, Tokyo, Japan). Colonoscopic findings were scored as follows: Mucosal thickening (0 = transparent, 1 = moderate, 2 = marked, and 3 = non-transparent), vasculature (0 = normal, 1 = moderate, 2 = marked, and 3 = absent/bleeding), and granularity (0 = none, 1 = moderate, 2 = marked, and 3 = extreme)^64^. Colonoscopy findings were scored by a blinded reviewer. For microscopic examination, colons were removed within 24 h of endoscopy after euthanizing the mice via cervical dislocation under anesthesia with isoflurane (Viatris). Distal colons (3 cm) were longitudinally cut, fixed with 10% neutral-buffered formalin (FUJIFILM Wako Pure Chemical, Osaka, Japan), embedded in paraffin wax, and stained with hematoxylin and eosin (H&E). Tissue specimens for pathological evaluation were prepared by the Biopathology Institute (Oita, Japan). Distal colons were scored according to the histological criteria as follows: Inflammatory infiltration (0 = none, 1 = < 10% mucosal infiltration, 2 = 10–25% mucosal infiltration and little submucosal infiltration, 3 = 26–50% submucosal infiltration, and 4 = > 50% transmural infiltration), epithelial hyperplasia (0 = none, 1 = < 1.25-fold increase in epithelial length, 2 = 1.25–1.35-fold increase in epithelial length, 3 = 1.36–1.5-fold increase in epithelial length, and 4 = > 1.5-fold increase in epithelial length), goblet cell and crypt loss (0 = none, 1 = < 20%, 2 = 20–35%, 3 = 36–50%, and 4 = > 50%), cryptitis (0 = none and 2 = presence), and crypt abscess (0 = none and 3 = presence)^41^. Histological scoring was supported by KAC (Kyoto, Japan) under blinded conditions.

### Measurement of plasma antibody levels

Plasma samples were stored at − 80 °C until use. Plasma IgG and IgA levels were determined using ELISA kits (Bethyl Laboratories, Montgomery, TX, USA), according to the manufacturer’s instructions. Levels of pANCA (anti-MPO IgG) and anti-flagellin IgG antibodies were detected by ELISA. Briefly, diluted plasma samples and standards (anti-MPO IgG (Abcam, Cambridge, UK) or anti-flagellin IgG (InvivoGen, San Diego, CA, USA)) were added to wells pre-coated with recombinant mouse MPO (R&D Systems, Minneapolis, MN, USA) or flagellin (FLA-ST; InvivoGen) and incubated at room temperature for 2 h. After washing with PBS/Tween 20, diluted horseradish peroxidase-conjugated anti-mouse IgG (Thermo Fisher Scientific, Waltham, MA, USA) was added to each well and incubated for 2 h. After washing with PBS/Tween 20, TMB substrate was added to each well and incubated in the dark. Subsequently, the stop solution was added to each well, and absorbance was measured at 450 nm.

### Isolation of colonic LP cells

LP cells were isolated from the colon, as previously described^64^. Briefly, distal colons were longitudinally cut, washed with cold Hank’s balanced salt solution containing 2% fetal bovine serum (FBS), penicillin (100 U/mL), and streptomycin (100 U/mL), and minced. The tissues were completely digested at 37 °C for 40 min with gentle stirring in the RPMI-1640 medium containing 2% FBS, penicillin (100 U/mL), and streptomycin (100 U/mL) and supplemented with collagenase D (100 U/mL; Sigma-Aldrich, St Louis, MO, USA) and DNase (20 mg/mL; Roche Diagnostics, Basel, Switzerland). LP cells were purified on a 40/75% Percoll gradient via centrifugation at 600 × *g* for 20 min at 25 °C.

### Flow cytometry

Antibodies against CD45, TCRβ, CD11b, B220, CD19, CD138, IgA, IgG2a/b and IgG3 were purchased from BD Biosciences (San Jose, CA, USA). Antibodies against CD4, CD8b, CD11b, CD11c, F4/80, Ly6G, Ly6C, CD335 (NKp46) and IgG1 were purchased from BioLegend (San Diego, CA, USA). The details of all antibodies used in this study, including fluorochrome conjugates, manufacturers, and catalog numbers, are provided in Table 1. Cell surface staining was performed according to standard techniques after treatment with the anti-CD16/32 antibody (BD Biosciences) to block FcγR binding. Dead cells were excluded using the Fixable Viability Dye eFluor 780 (FVD780; Thermo Fisher Scientific). For intracellular staining, the cells were stained with different cell surface antibodies, fixed, permeabilized, and intracellularly stained for IgA, IgG1, IgG2a/b, or IgG3. Gating strategies were set with reference to the isotype or fluorescence minus one control. Flow cytometry was performed using the BD LSRFortessa X-20 flow cytometer (BD Biosciences), and data were analyzed using the FlowJo version 10.8.0 software (BD Biosciences).

**Table 1:** List of antibodies used for flow cytometry analysis.

Antigen	Fluorochrome	Manufacturer	Catalog number
CD45	Brilliant UV395	BD Biosciences	564,279
TCRβ	Brilliant Blue 700	BD Biosciences	745,846
CD11b	Brilliant Violet 510	BioLegend	101,263
CD11b	Alexa Fluor 700	BD Biosciences	557,960
Ly6G	Brilliant Violet 711	BioLegend	127,643
Ly6G	PE/Cyanine7	BioLegend	127,618
F4/80	Brilliant Violet 785	BioLegend	123,141
CD11c	PE	BioLegend	117,308
Ly6C	Pacific Blue	BioLegend	128,014
CD335	PE/Dazzle 594	BioLegend	137,630
CD8b	Alexa Fluor 700	BioLegend	126,618
CD4	Brilliant Violet 605	BioLegend	100,548
B220	Brilliant UV737	BD Biosciences	612,838
CD19	PE	BD Biosciences	561,736
CD138	Brilliant Violet 421	BD Biosciences	562,610
IgA	FITC	BD Biosciences	559,354
IgG1	APC	BioLegend	406,609
IgG2a/b	Brilliant Violet 510	BD Biosciences	744,293
IgG3	Brilliant Violet 650	BD Biosciences	744,136

Details of the antibodies employed for flow cytometric analysis are summarized in this table, including their target antigens, conjugated fluorochromes, manufacturers, and catalog numbers.

### Immunohistochemistry for IgG

For CD19 and IgG staining, deparaffinized and rehydrated sections were treated with a protease solution (Nichirei Biosciences, Tokyo, Japan) for 15 min at 37 °C and incubated with Blocking One (Nacalai Tesque) for 1 h at room temperature. Then, the sections were incubated with Alexa Fluor 488-conjugated anti-CD19 (BioLegend) and Alexa fluor 555-conjugated anti-IgG (SouthernBiotech, Birmingham, AL, USA) at 4 °C overnight. Nuclei were counterstanied using 4’,6-diamidino-2-phenylindole (DAPI; SouthernBiotech). The sections were mounted using Fluoromount-G (SouthernBiotech) and sequentially scanned using the FV3000 confocal laser-scanning microscope (Olympus) for double-positive cells. For IgG deposits, the sections were mounted using Fluoromount-G (SouthernBiotech) and sequentially scanned using the BZ-X800 fluorescence microscope (Keyence, Osaka, Japan). Microscopic images were digitized, and IgG-positive areas were determined by red–green–blue (RGB) segmentation. Digital images were analyzed using inform version 3.1.2 software (PerkinElmer, Hopkinton, MA, USA). Results were calculated as the number of IgG-positive areas in the mucosa and submucosa in all fields of the distal colon.

### Colonic fibrosis analysis

Masson’s trichrome staining was performed to measure the collagen deposition as fibrosis. The tissue specimens were prepared for pathological evaluation at the Biopathology Institute. Microscopic images were digitized, and collagen-positive areas were determined by RGB segmentation. Digital images were analyzed using the inform version 3.1.2 software (PerkinElmer). Results were calculated as the number of collagen-positive areas in the mucosa and submucosa in all fields of the distal colon. For αSMA staining, deparaffinized and rehydrated sections were treated with HISTOFINE (pH 9.0; Nichirei Biosciences) for 15 min at 95 °C and incubated with Blocking One (Nacalai Tesque) for 30 min at room temperature. Then, αSMA staining was performed using rabbit anti-αSMA (Abcam) at 4 °C overnight, followed by incubation with Alexa Fluor 647 anti-rabbit IgG (Abcam) at room temperature for 1 h. Nuclei were counterstained with DAPI (SouthernBiotech). The sections were mounted using Fluoromount-G (SouthernBiotech) and sequentially scanned using the BZ-X800 fluorescence microscope (Keyence).

### RNA isolation and real-time PCR

Total RNA was isolated from the distal colonic tissues using the RNeasy Mini Kit (Qiagen, Hilden, Germany) according to the manufacturer’s instructions. RNA samples were quantified using an ND-1000 spectrophotometer (NanoDrop Technologies, Wilmington, DE, USA). Quantitative RT-PCR was performed using the RNA-to-CT 1-step kit (Thermo Fisher Scientific) and QuantStudio 7 Flex Real-Time PCR System (Thermo Fisher Scientific). All primers (*TGFβ*: Mm01178820_m1 and *glyceraldehyde 3-phosphate dehydrogenase* (*GAPDH*): Mm99999915_g1) were purchased from Thermo Fisher Scientific. The threshold cycle (Ct) generated by quantitative RT-PCR system was used, and the mRNA expression levels were normalized to the level of GAPDH expression according to the 2^−ΔΔCt^ method.

### Measurement of colonic cytokine levels

Distal colonic segments were rinsed with saline, blotted dry, and stored at − 80 °C. The segments were homogenized using beads in DW containing the Sample Diluent Concentrate 1 (R&D Systems) and protease inhibitor cocktail (Sigma-Aldrich). After centrifugation at 12,000 × *g* for 10 min at 4 °C to remove the debris, protein concentration was determined using the DC protein assay kit (Bio-Rad Laboratories, Richmond, CA, USA). Colonic cytokine levels were quantified using the Meso Scale Discovery electrochemiluminescence assay for TNF-α, IL-6, and IL-1β (Meso Scale Diagnostics, Rockville, MD, USA), according to the manufacturer’s instructions.

### Measurement of NF-κB transcriptional activity in the colon

NF-κB activity in the colon was determined using the TransAM transcriptional factor assaying kit for NF-κB p65 (Active Motif, Carlsbad, CA, USA), according to the manufacturer’s instructions. NF-κB p65 protein (Active Motif) was used as the standard.

### Statistical analyses

Significance of the differences between two groups was evaluated using the F-test, followed by the Student’s or Aspin–Welch *t*-test. Differences in disease severity were evaluated using the Mann–Whitney U test.

## Supplementary Information

Below is the link to the electronic supplementary material.

Supplementary Material 1

## Data Availability

The data that support the findings of this study are available from the corresponding author upon reasonable request.
