# Supplementary material for: Depletion of IgG-producing plasma cells in the colon by treatment of proteasome inhibitor ameliorates chronic DSS-induced colitis in mice
Source: Sci Rep. 2026 Jan 8;16:4657. doi: 10.1038/s41598-025-34868-6 (PMC12867971; doi:10.1038/s41598-025-34868-6)
Supplement: Supplementary file 1 — Supplementary Material 1 [file 41598_2025_34868_MOESM1_ESM.pdf]

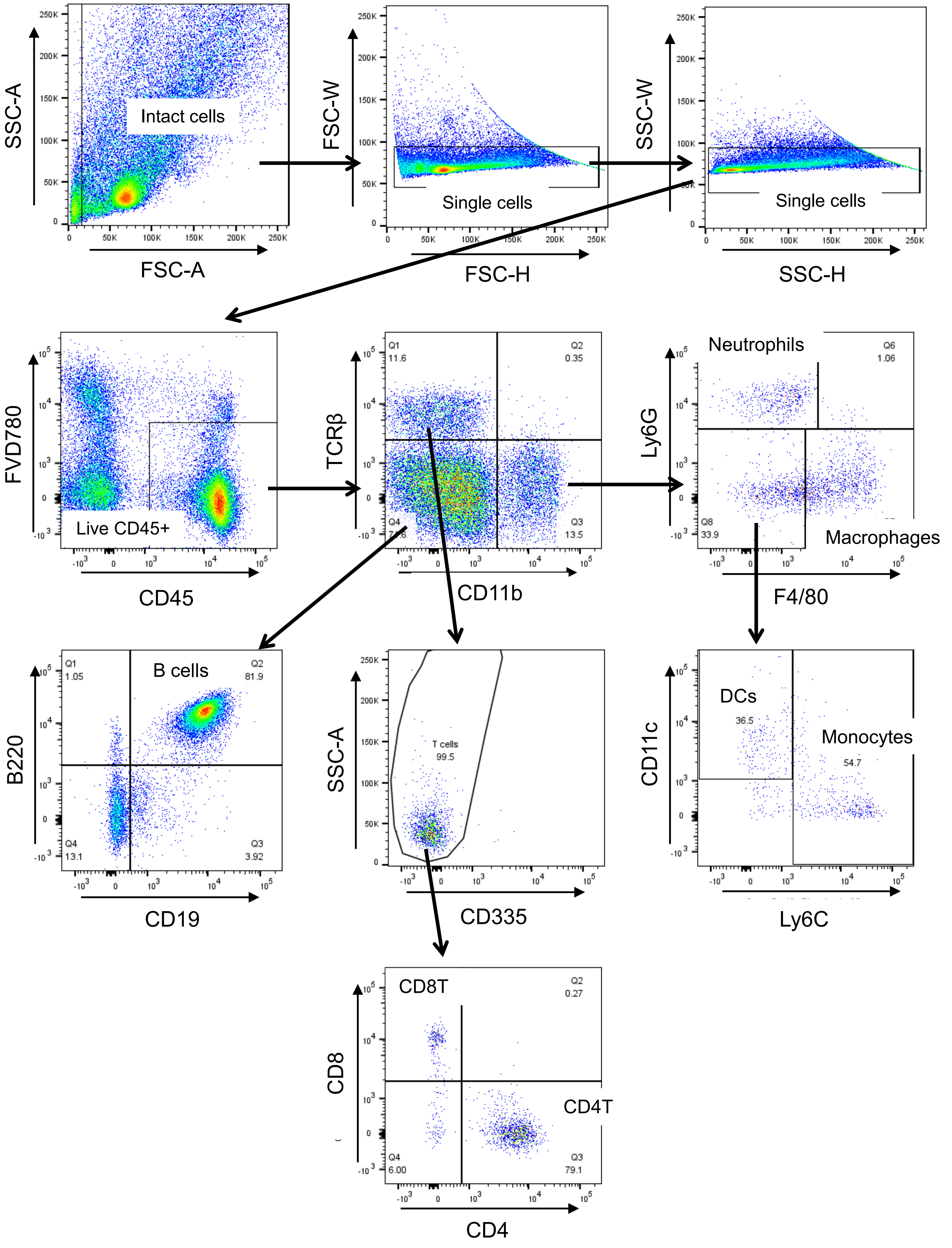

Supplementary Figure S1

Gating strategy for the analysis of colonic LP cells

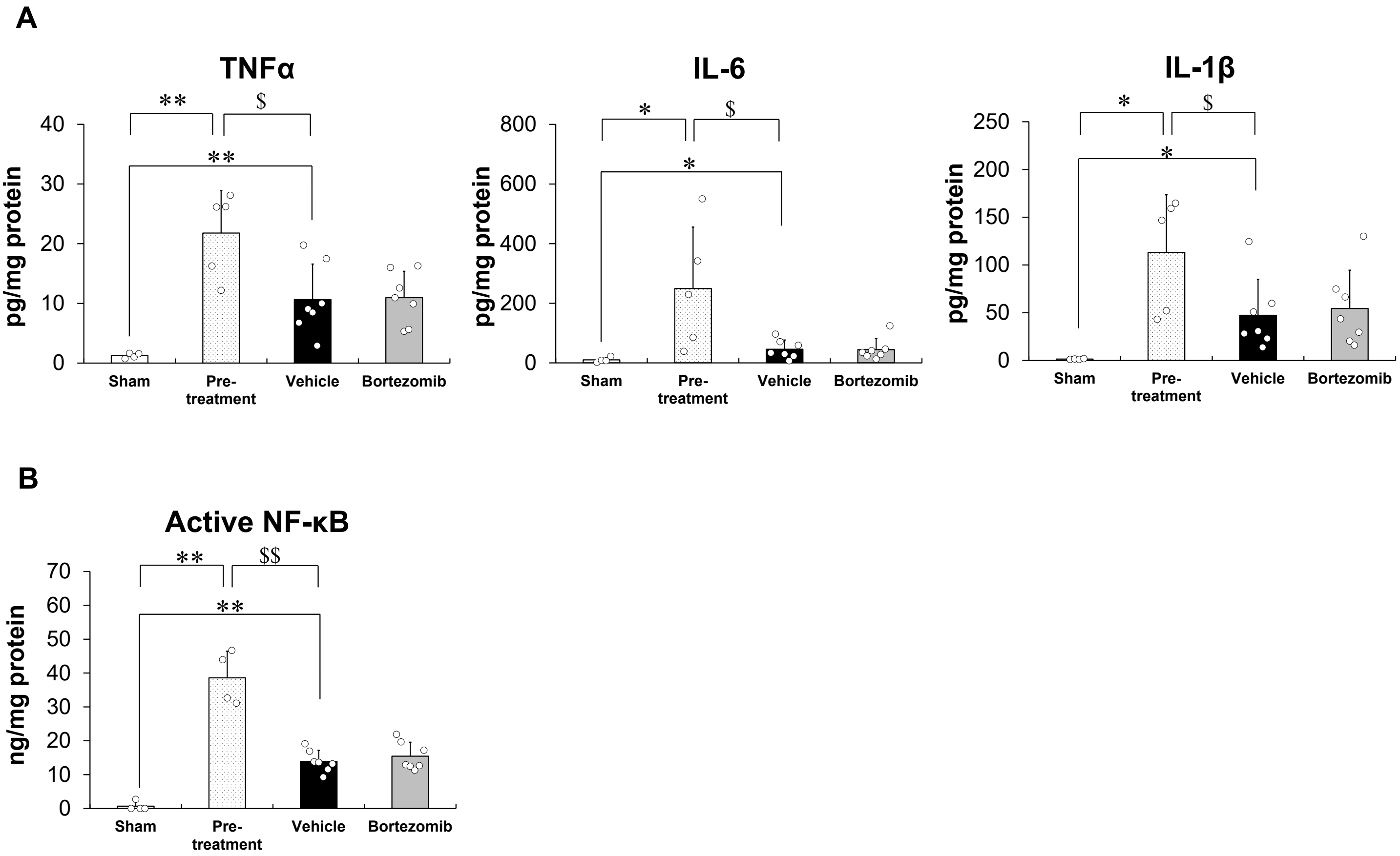

Supplementary Figure S2

Effects of bortezomib on inflammatory cytokine production and NF-κB activation

A) Inflammatory cytokine levels in the colon tissues of the chronic DSS-induced colitis model. ELISA kits were used to measure the tumor necrosis factor (TNF)-α, IL-6, and IL-1β levels in the colon tissues of mice.

B) NF-κB activation in the colon tissues of the chronic DSS-induced colitis model.

Results are shown as the mean ± SD with individual data. n = 4–7 animals per group.
*p < 0.05 and **p < 0.01 vs. sham-treated group; §p < 0.05 and §§p < 0.01 vs. pre-treatment group determined by Student’s or Aspin–Welch t-test performed

A

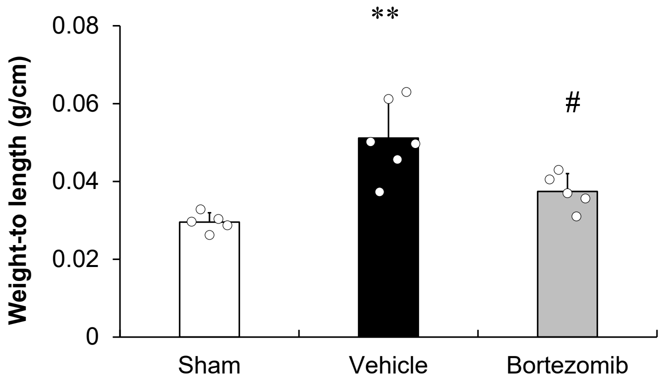

B

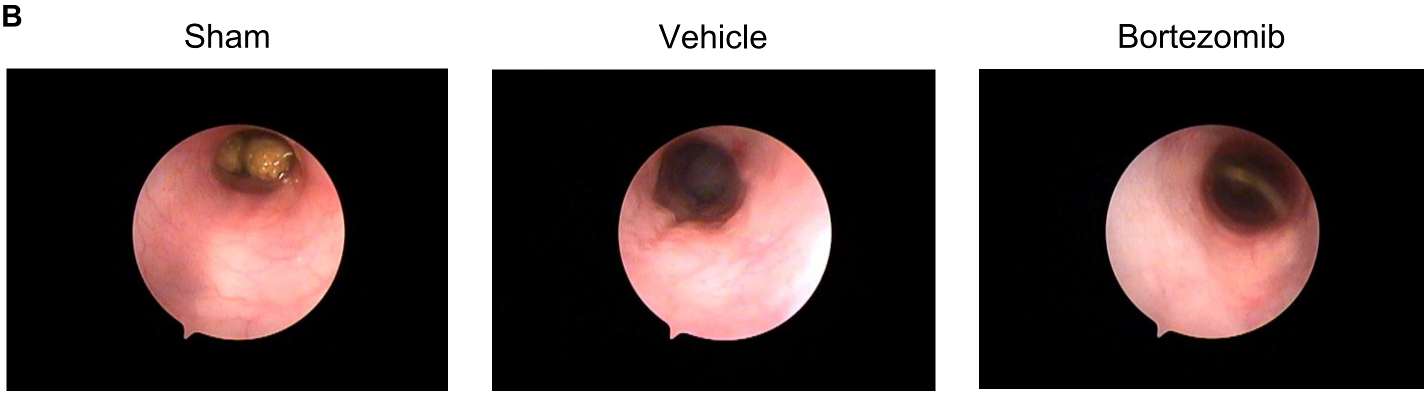

C

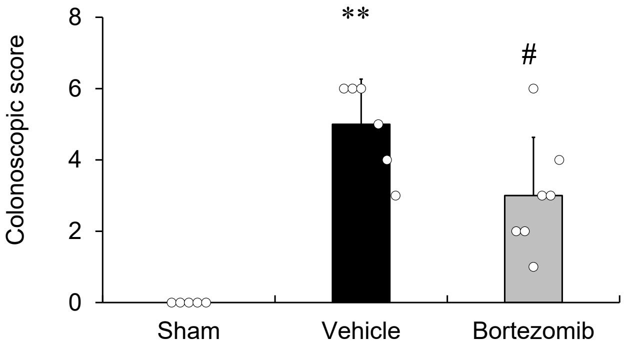

Supplementary Figure S3

Effects of bortezomib on the progression of colitis.

A) Distal colon weight-to-length ratio at the experimental endpoint as g/cm. Results are shown as mean ± SD with individual data. **p < 0.01 vs. sham-treated group, #p < 0.05 by Student's t-test performed vs. vehicle-treated group (Student's or Aspin–Welch t-test). n = 5–7 animals per group.

B) Representative colonoscopic pictures of the colon tissues of sham-, vehicle-, or bortezomib-treated mice.

C) Colonoscopic scores of sham-, vehicle-, or bortezomib-treated mice at the experimental endpoint. Results are shown as mean ± SD with individual data. **p < 0.01 vs. sham-treated group, #p < 0.05 vs. vehicle-treated group determined by Mann-Whitney U test. n = 5–7 animals per group.

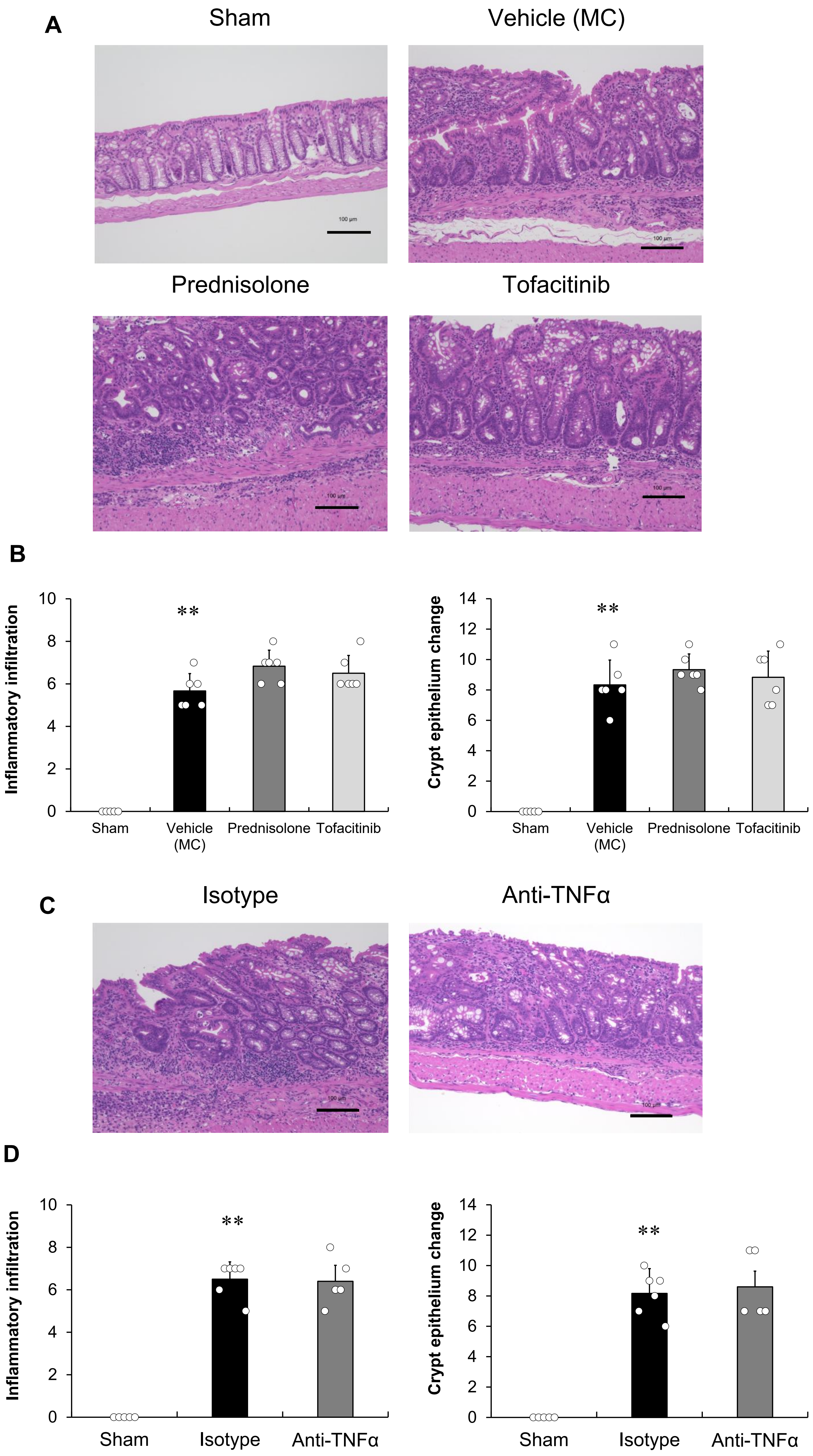
A
Sham
Vehicle (MC)
Prednisolone
Tofacitinib
100 μm
B
Inflammatory infiltration
0
2
4
6
8
10
**
Sham
Vehicle (MC)
Prednisolone
Tofacitinib
Crypt epithelium change
0
2
4
6
8
10
12
14
**
Sham
Vehicle (MC)
Prednisolone
Tofacitinib
C
Isotype
Anti-TNFα
100 μm
D
Inflammatory infiltration
0
2
4
6
8
10
**
Sham
Isotype
Anti-TNFα
Crypt epithelium change
0
2
4
6
8
10
12
14
**
Sham
Isotype
Anti-TNFα

Supplementary Figure S4

Effects of anti-inflammatory drugs on the progression of colitis.

A) Representative microphotographs of the colonic tissues of sham-, vehicle (MC)-, tofacitinib (100 mg/kg)-, or prednisolone (3 mg/kg)-treated mice (H&E stain). Scale bar, 100 μm.

B) Histological score at the experimental end point. Results are shown as mean ± SD with individual data. **$p < 0.01$ vs. sham-treated group determined by Mann – Whitney U test.

C) Representative microphotographs of the colonic tissues of Isotype- or anti-TNFα antibody-treated mice (H&E stain). Scale bar, 100 μm.

D) Histological score at the experimental end point. Data are shown as mean ± SD with individual data. **$p < 0.01$ vs. sham-treated group determined by Mann–Whitney U test.
